# Editorial: Insights into the molecular studies of plant growth-promoting microorganisms for sustainable agricultural production

**DOI:** 10.3389/fmicb.2023.1218652

**Published:** 2023-06-13

**Authors:** Naeem Khan, Noshin Ilyas, Robert Czajkowski, KOTB Attia

**Affiliations:** ^1^Department of Agronomy, University of Florida, Gainesville, FL, United States; ^2^Department of Botany, PMAS-Arid Agriculture University, Rawalpindi, Pakistan; ^3^Intercollegiate Faculty of Biotechnology UG and MUG, University of Gdansk, Gdansk, Poland; ^4^Center of Excellence in Biotechnology Research, King Saud University, Riyadh, Saudi Arabia

**Keywords:** environmental stresses, sustainable agriculture, regulation of plant defense system, gene expression, host microbe interactions

Plant growth-promoting rhizobacteria (PGPR) are group of bacteria inhabiting the rhizosphere and colonize the root environment. These organisms can be used for improving plant growth and for the sustainability of agricultural production under unfavorable environments. Rhizosphere microorganisms can produce extracellular chemical signals that help in establishing a complex signaling network between host and microbes. PGPR colonizes plant roots, inspires plant growth, and reduces disease or injury due to insects. Presently abundant research work has been done on PGPR and many of them are being commercialized for crops. PGPR can be used as bio-fertilizers for the improvement of plant health and yield under stressful environments. Bio-fertilization is considered as a major nitrogen source for different crop plants worldwide. Similarly, PGPR are accountable for increasing N-fixation in legumes, promote free-living N-fixing bacteria, and improve the availability and distribution of supplementary nutrients in the rhizosphere (Daniel et al., 2022). They are also responsible for the phytohormones production thus play a critical role in plant normal growth and development. PGPR decreases the inhabitants of root pathogens and other harmful microorganisms in the rhizosphere, thus promoting plant growth.

Microorganisms are involved in the alteration of metabolomic pathways of the host plants thus assisting in systemic resistance of the plants. They help in the up-regulation of stress-responsive secondary metabolites, thus helping in the regulation of metabolic activities in cells. However, a better insight into their mechanism of action and their starring role in plant growth and development is essential for agricultural production and research. It has been investigated that *Rhodopseudomonas palustris* strain PS3 enhanced the growth and yield of tomato plants. The strain also substantially improved the soil nutrient content and the quality of tomato fruit. These favorable bacterial communities effectively contribute to the improvement of fruiting and yield and soil health (Lee et al.).

Microorganisms play a significant role in the bioremediation of heavy metals. The accumulation of heavy metals in the food crops not only reduces the growth and yield of crop plants but also has harmful impacts on human health. Recently it has been reported that the collective application of fungal strains *Stemphylium lycopersici* and *Stemphylium solani* significantly reduced the adverse effects of chromium stress in soybean by converting the toxic Cr-VI to a less toxic form known as Cr-III. The isolated fungal strains also boosted the growth of soybean under heavy metal stress by enhancing the production of phytohormones, plant secondary metabolites, antioxidant enzymes and free radical scavenging enzymes. Based on these results, both the fungal isolates can be used as a potential candidate for developing a biofertilizer for Cr contaminated soils (Husna et al.).

Metabolomics is utilized as a principal tool to comprehend the responses of plants to various biotic and abiotic factors. Several biogenic volatile compounds are produced in different plant species. However, little information is available about the contribution of PGPR in altering the plant metabolome and making them more resistant to various stresses (Khan et al., 2019). A current study reported the impacts of PGPR on the metabolome of above ground tissues. The results revealed the differential accumulation of different classes of plant metabolites; some of those were reported to have a role in plant microbe interactions. The metabolic perturbations correlated with the different metabolites were observed in the leaves of PGPR-bio-primed plants, suggesting the role of PGPR in altering the plant metabolome. The results of this study provide a clear insight that PGPR seed biopriming can induce changes in plant metabolomes leading to induced systemic resistance against biotic and abiotic stresses. The study led to a better understanding of PGPR induced metabolites in plants and their role in plant growth, stress tolerance and in sustainability of agriculture (Mashabela et al.). It has also been reported that the application of *P. aeruginosa* hinders the mechanisms of quorum-sensing in soft rot pathogen *Lelliottia amnigena* by generating specific metabolites accountable for the inhibition of quorum sensing. The ethyl acetate extract of the same bacterium was inhibitory to biofilm formation thus suppressed the pathogenicity related traits of soft rot initiating pathogen *L. amnigena*. This study is a useful step toward the development of a novel strategy to control the plant pathogen virulence in susceptible plants (Kapadia et al.). Lin et al. reported that the application of phosphate solubilizing strain *Bacillus megaterium* upregulated the expression of genes responsible for drought, salt and heat stress in plants. The strain also effectively upregulated the amino acids and carbohydrate metabolic pathways in potato plants. Beside this the strain helps in absorption of nutrients, promoting root growth, physiological, and metabolic activities in potato plants. The use of PGPR in agriculture offers a promise for improving the growth of plants and their resistance to various stresses by altering plant metabolomes.

## Author contributions

NK wrote the editorial. NI, RC, and KA reviewed and edited the editorial. All authors contributed to the article and approved the submitted version.
